# Offline crime bounces back to pre-COVID levels, cyber stays high: interrupted time-series analysis in Northern Ireland

**DOI:** 10.1186/s40163-021-00162-9

**Published:** 2021-11-10

**Authors:** David Buil-Gil, Yongyu Zeng, Steven Kemp

**Affiliations:** 1 Department of Criminology, School of Social Sciences, University of Manchester, 2.17 Williamson Building, Oxford Road, Manchester, M15 6FH UK; 2 Law School, Lancaster University, Lancaster, UK; 3 Department of Law, Pompeu Fabra University, Barcelona, Spain

## Abstract

Much research has shown that the first lockdowns imposed in response to the COVID-19 pandemic were associated with changes in routine activities and, therefore, changes in crime. While several types of violent and property crime decreased immediately after the first lockdown, online crime rates increased. Nevertheless, little research has explored the relationship between multiple lockdowns and crime in the mid-term. Furthermore, few studies have analysed potentially contrasting trends in offline and online crimes using the same dataset. To fill these gaps in research, the present article employs interrupted time-series analysis to examine the effects on offline and online crime of the three lockdown orders implemented in Northern Ireland. We analyse crime data recorded by the police between April 2015 and May 2021. Results show that many types of traditional offline crime decreased after the lockdowns but that they subsequently bounced back to pre-pandemic levels. In contrast, results appear to indicate that cyber-enabled fraud and cyber-dependent crime rose alongside lockdown-induced changes in online habits and remained higher than before COVID-19. It is likely that the pandemic accelerated the long-term upward trend in online crime. We also find that lockdowns with stay-at-home orders had a clearer impact on crime than those without. Our results contribute to understanding how responses to pandemics can influence crime trends in the mid-term as well as helping identify the potential long-term effects of the pandemic on crime, which can strengthen the evidence base for policy and practice.

## Introduction

The COVID-19 pandemic and the first stay-at-home orders imposed by national and regional governments were linked to notable decreases in some types of violent and property crime in the United States (Abrams, 2021; Ashby, 2020; Mohler et al., 2020), the United Kingdom (Halford et al., 2020) and other countries (Nivette et al., 2021). Simultaneously, research observed increases in other offences that occur in physical and digital places affected differently by lockdown mobility restrictions, such as domestic violence (Piquero et al., 2021), cyber-enabled fraud (Kemp et al., 2021), online hate speech (Stechemesser et al., 2020) and some forms of hacking (Buil-Gil et al., 2021). It appears that the initial social distancing measures contributed to a reduction in opportunities for offenders to physically converge with crime targets in outdoor urban areas, but they also had the unintended consequence of increasing the presence of crime targets and offenders on the internet.

After the first few months of the COVID-19 pandemic, researchers noted that much traditional, offline crime had begun to bounce back to pre-COVID levels (Balmori de la Miyar et al., 2021; Langton et al., 2021; Nix & Richards, 2021), and some violent offences surpassed crime rates seen before the pandemic (Kim & Phillips, 2021). People were returning to the streets, and consequently opportunities for offenders to find suitable crime targets in outdoor spaces were returning to normal levels. However, there is a lack of research on the medium- and long-term impact of multiple lockdown orders on cyber-enabled and cyber-dependent crime. More importantly, crime research has yet to understand whether the peak rates in cybercrime seen immediately after the first lockdown orders returned to pre-COVID levels after the easing of stay-at-home restrictions, or whether cybercrime rates remained above pre-pandemic trends, thus indicating a potential long-term post-pandemic upward trend in cybercrime. For instance, the increase in certain online activities brought about by COVID-19 could contribute to long-lasting changes in routine activities on the internet (e.g., teleworking, online shopping), and changes in online crime trends may extend beyond the pandemic. Furthermore, there have been few attempts to compare online and offline crime in the same dataset, thereby limiting comparisons of trends between these crime types.

In this study, we analyse changes in crime, including offline and online crime, in Northern Ireland during COVID-19, and investigate the short- and medium-term impact of the three lockdowns on crime. We analyse the effect of multiple lockdowns on crime using interrupted time series (ITS) analysis based on segmented linear regressions and counterfactuals (McDowall et al., 2019).

## COVID-19 and changes in everyday life in Northern Ireland

**Fig. 1:**
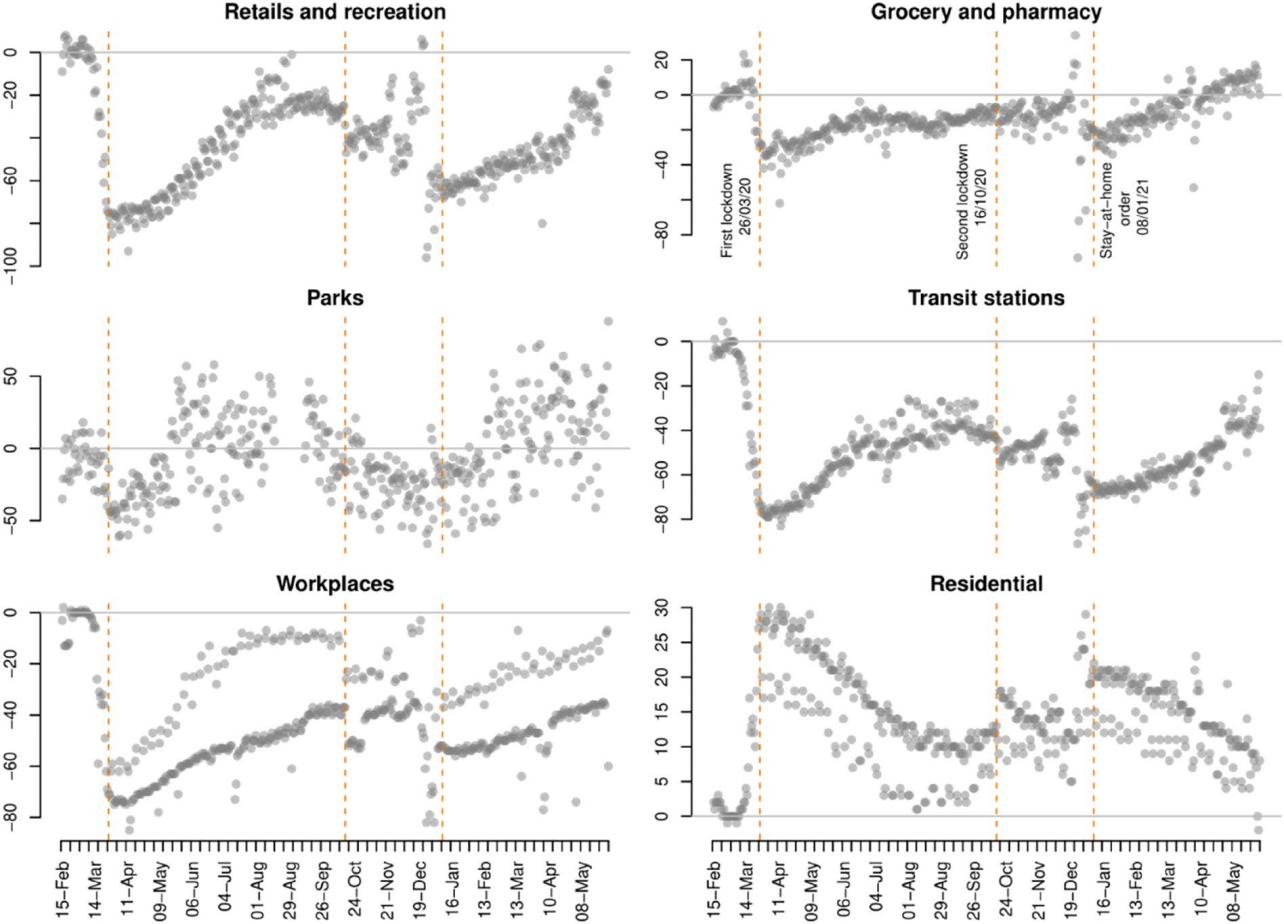
Percent change from baseline in mobility indicators in Belfast (February 15th, 2020, to May 31st, 2021). Source: Google COVID-19 Community Mobility Reports (https://www.google.com/covid19/mobility/index.html?hl=en)

The timeline of the COVID-19 pandemic in Northern Ireland was similar to other parts of the UK. The first case of COVID-19 was detected on February 27th, 2020, and the number of cases rose steeply throughout March. In order to control the spread of the virus, the UK Government announced the first national lockdown on March 23rd, which came into force on March 26th. The stay-at-home order meant all non-essential social and business activities were restricted for weeks, and non-essential shops, schools, universities, businesses, pubs and other venues were closed. These measures had enormous effects on mobility trends, as can be seen in Fig. 1, with almost immediate reductions in mobility in places dedicated to retail and recreation, grocery and pharmacy, transit stations and workplaces, and marked increases in mobility in residential areas. The first lockdown was gradually eased during June and July 2020.

Due to the steep rise in COVID-19 infections during late September and early October 2020, the Northern Ireland Government announced a second lockdown on October 14th, which officially began on October 16th. This second lockdown involved the closure of schools, universities and the hospitality sector, but it did not involve a stay-at-home order as such and the social distancing restrictions were less strict than in the first national lockdown. Although the measures associated with the second lockdown contributed to immediate changes in mobility (see Fig. 1), the extent of these changes was relatively small. Further restrictions, mostly related to the closure of cafes, hospitality, non-essential shops and gyms, were introduced on November 27th. The second lockdown was mostly lifted by the second week of December.

Just a few days later, on December 17th, a third lockdown was announced, which began on December 26th. Entertainment and hospitality businesses and non-essential shops were closed, and a maximum of three households were allowed to meet over Christmas. Some mobility restrictions were later tightened on January 8th, 2021, when a stay-at-home order came into force due to the spread of a new variant of the virus. People were only allowed to leave home for medical reasons, to buy food, exercise and go to work only when work could not be done from home. As can be seen in Fig. 1, some of these measures had a similar impact on mobility as the first lockdown (some of the extreme changes in mobility seen during the last days of December are due to Christmas shopping and celebrations). Stay-at-home orders were progressively lifted during March and April 2021, and mobility trends progressively returned to the pre-COVID baseline.

These unprecedented changes in routine activities brought about by the COVID-19 lockdowns are expected to have short- and medium-term impacts on crime in Northern Ireland, as seen in other parts of the world (Nivette et al., 2021). We will analyse changes in crime after the first lockdown (March 23rd, 2020), second lockdown (October 16th, 2020) and the stay-at-home order of the third lockdown (January 8th, 2021).

## Rapid social changes and crime: the COVID-19 case

Crime is dependent on illicit opportunity structures which vary according to changes in everyday routine activities. At the end of the 1970s, Cohen and Felson (1979) observed that property and violent crime was growing in the United States due to a series of social changes that increased the availability of suitable targets and reduced the ability of people to serve as guardians of these targets. Cohen and Felson (1979) proposed the Routine Activity Approach of crime, which argues that crime increases when (and where) there are more opportunities for offenders to converge with suitable targets in the absence of capable guardians. Since then, this approach has been applied to explain the effect of natural disasters on crime (Leitner et al., 2011), the impact of rapid economic and political changes on crime (Piatkowska et al., 2016), and changes in crime during large sport events (Kalist and Lee, 2016), amongst many other examples. However, no event in recent history has affected everyday routine activities as much as COVID-19 and the associated lockdown measures.

After the first COVID-19 lockdown was announced in many countries in March 2020, several researchers noted immediate changes in crime. Mohler et al. (2020) observed that burglary and robbery reports decreased after the first stay-at-home order in Los Angeles and Indianapolis. Also using Los Angeles crime data, Campedelli et al. (2021) observed a significant decrease in robbery, shoplifting, theft and battery during March and April 2020, but no significant changes were seen for burglary, homicide, vehicle theft or assault. Ashby (2020) analysed crime data in sixteen large US cities between January and May 2020 and noted a reduction in residential burglary and motor vehicle theft in some cities after the first stay-at-home orders. There was little variation in non-residential burglary and serious assault. In the UK, Halford et al. (2020) analysed changes in crime in Lancashire during March 2020, and noted that, by the week of March 23rd, there had been a large decrease in shoplifting, theft, theft from vehicles, domestic abuse, assault and residential and non-residential burglary. Changes in recorded crime were also found in Sweden (Gerell et al., 2020), Mexico (Estévez-Soto, 2021), China (Borrion et al., 2020) and Australia (Payne et al., 2021). Nivette et al. (2021) recorded crime data from 27 cities across 23 countries and concluded that stay-at-home orders contributed to a considerable global drop in urban crime. Social distancing measures imposed to control the spread of the virus reduced the presence of suitable targets and offenders on the streets, thus contributing to an immediate reduction in opportunities for many types of property and violent crimes in outdoor areas.

Some researchers noted, however, that while many types of offline crime were decreasing, there were signs that the changes in routine activities brought about by the first lockdown had increased opportunities for online crime. Using data about cyber-enabled fraud and cyber-dependent crime recorded by Action Fraud, the UK National Fraud and Cybercrime Reporting Centre, between May 2019 and May 2020, Buil-Gil et al. (2021) observed significant increases in some forms of hacking and online shopping fraud after the first stay-at-home order. Lallie et al. (2021) documented cyber-attacks reported globally and observed an increase in cybersecurity incidents such as phishing, malware and cyber-enabled fraud after February 2020. Kemp et al. (2021) analysed reports of fraud and cybercrime made to Action Fraud UK and observed a large increase in cyber-dependent crime (i.e., hacking, denial of service attacks and malware), online shopping fraud and dating fraud after the first COVID-19 lockdown, while those forms of fraud associated with offline events, such as doorstep fraud and ticket fraud, decreased. As argued by these researchers, the first stay-at-home orders contributed to an immediate spike in internet use for entertainment, teleworking, socialising, shopping, and meeting new people, thereby increasing the amount of valuable crime targets in online environments. Other forms of crime enabled by the internet also increased, for example, Stechemesser et al. (2020) recorded Tweets with anti-Chinese racist content and observed a spike in online hate speech during March 2020.

This body of literature contributes to understanding the effect of pandemic-induced, large-scale, rapid social changes on offline and online crime. However, crime research is not only interested in the short-term impact of the COVID-19 lockdown on crime, but it also aims to understand the effect of stay-at-home orders on medium- and long-term crime trends. Langton et al. (2021) showed that after the first COVID-19 lockdown in the UK, crime started to bounce back to pre-COVID levels. Opportunities for offenders to find suitable targets on the street returned to normal when social distancing measures were relaxed. Similar results were found by Balmori de la Miyar et al. (2021) using data recorded in Mexico. Nix and Richards (2021) also observed that domestic violence calls for police services in the US returned to pre-COVID levels when lockdown restrictions were lifted.

Existing research appears to indicate that the quick changes in offline crime seen immediately after the first COVID-19 lockdown were temporary, and crime trends progressively returned to pre-COVID levels after social distancing restrictions were relaxed. Nonetheless, while some of the changes in offline routine activities brought about by stay-at-home orders may indeed be temporary (e.g., bars and restaurants reopen, employees return to work from the office, sport events and concerts are organised, travelling is allowed), some of the changes in online everyday practices may not be restricted to the pandemic and may have long-term effects on cybercrime. Online shopping is a clear example, since internet sales were well above pre-COVID levels even after May 2021 (Office for National Statistics, 2021). There is also expected to be a long-term post-pandemic upward trend in use of online gaming, social media, teleworking, online food delivery, online conference platforms and online dating (Nurse et al., 2021; Ofcom, 2021). Thus, it is plausible that the rising trend seen in cybercrime since March 2020 may not return to levels recorded before the pandemic.

## Methodology

### Data

In this article we analyse data recorded by the Police Service of Northern Ireland between April 2015 and May 2021. Crime data was accessed from the crime open data portal.^1^ To our knowledge, the Police Service of Northern Ireland is the only UK police force that publishes open access crime data for both offline and online offences, thus allowing us to analyse the impact of the first, second and third lockdowns on both crime types. We analyse the following types of crime aggregated by month: (a) violence and sexual crime, (b) drug crimes, damage and public order, (c) burglary, (d) theft and (e) fraud and cybercrime.^2^ Open access fraud and cybercrime data is only available at the monthly level, and therefore we analyse changes in crime across months.

We analyse a variety of crimes that could be affected in different ways by the mobility restrictions of the three lockdowns. For example, opportunities for violent offences and theft are found mostly in ‘public places’ and thus were likely to decrease during stay-at-home orders and return to normal levels after each lockdown (Balmori de la Miyar et al., 2021). While residential burglary opportunities were likely to decrease during lockdown due to the increase of ‘capable guardians’ at home, this may not be the case for non-residential burglaries (Felson et al., 2020). Some fraud types are clearly cyber-enabled, such as online shopping fraud, and, therefore, opportunities were likely to grow with increased internet use both during and after lockdown, while other fraud categories may include both offline and online incidents (for example, investment and advance free fraud can be enabled by the internet in some cases but not always). Cyber-dependent crime can only take place online.

### Analytical approach

In order to analyse the immediate effect of each COVID-19 lockdown on crime, but also the medium-term changes in crime after each lockdown, we utilise ITS analysis based on segmented linear regressions (McDowall et al., 2019). The ITS segmented linear model used here is given by:

$$Y= {\beta }_{0}+{\beta }_{1}T+{\beta }_{2}{D}_{1}+{\beta }_{3}{P}_{1}+{\beta }_{4}{D}_{2}+{\beta }_{5}{P}_{2}+{\beta }_{6}{D}_{3}+{\beta }_{7}{P}_{3}+{\beta }_{8}S+e$$

where $Y$ is the value of crime in a given month, $T$ represents time (in months) from 1 to 74, ${D}_{1}$, ${D}_{2}$ and ${D}_{3}$ correspond to the first, second and third lockdowns, respectively, and ${P}_{1}$, ${P}_{2}$ and ${P}_{3}$ is the time (months) passed since the first, second and third lockdowns until the start of the next lockdown, respectively. The model also includes a dummy seasonal variable, $S$, to account for the changes in trends between spring–summer and autumn-winter for some crime types. In order to compare the observed crime trends with the expected changes in crime if COVID-19 had not happened, we calculate the ‘counterfactuals’ (i.e., the trends that crime would have followed if lockdown restrictions had not taken place). We predict the ‘counterfactuals’ from:

$$Y= {\beta }_{0}+{\beta }_{1}T+{\beta }_{2}S+e$$

Aside from a few exceptions (e.g., Humphreys et al., 2013; Steinbach et al., 2015), this approach has rarely been applied in crime research, but its application is widespread in epidemiology, economics and other fields.

While this simple approach enables us to obtain direct results to achieve our aims, it is not free from limitations. One of the main assumptions of the ordinary least squares (OLS) estimation used here is that error terms are independent from one another, but this may be highly problematic in time-series analysis when the score of *Y* (crime value) at one point in time is correlated with the scores at another points (i.e., there may be ‘serial autocorrelation’). In order to account for this threat to the validity of our results, we also estimate multivariate linear regressions with Auto Regressive Integrated Moving Average (ARIMA) errors as a sensitivity and robustness check.^3^ The results of the models with ARIMA errors are presented in the Appendix, showing markedly similar results to that of the ITS analysis, but we also note some differences that will be described in the next section. Marked differences across modelling approaches would indicate low robustness of results—and thus the need to be particularly cautious when we interpret our findings. While this sensitivity check indicates that our results are robust overall, in a few cases we identified some differences that will be described in detail. The analysis has been conducted in R software (R Core Team, 2021).

## Results

This section presents the results of the ITS analysis. The results of the multivariate ARIMA errors are presented in the Appendix as a sensitivity check.

**Fig. 2:**
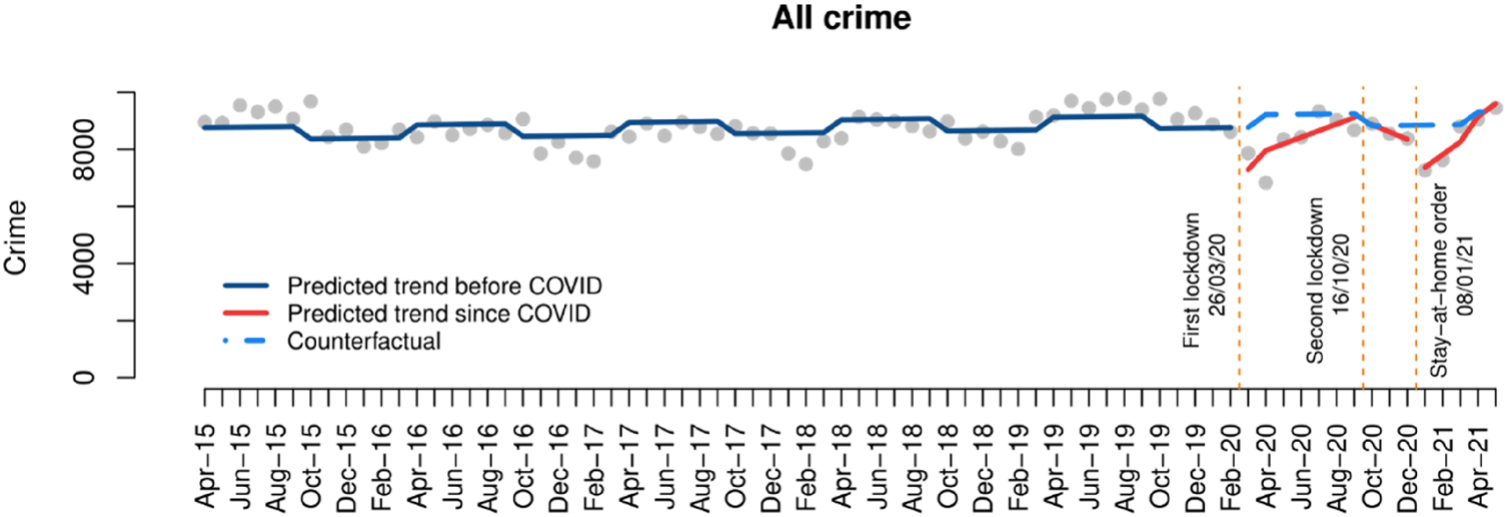
Interrupted time series analysis of all crime

Figure 2 shows the crime trend seen before COVID-19 (dark blue line), the predicted crime trend since March 2020 if COVID-19 had not taken place (i.e., the ‘counterfactual’, visualised with a dashed light blue line), and the actual trend observed after each lockdown (red lines). The same visualisation strategy will be used for each specific crime type in the following sections. Overall, recorded crime suffered a marked decrease after the first and third lockdowns in Northern Ireland, while the effect of the second lockdown was less evident. Crime rates progressively returned to pre-COVID levels after the first and third lockdown. However, as described in the literature review, this is likely to mask substantial differences between crime types.

### Violence and sexual crime

**Fig. 3:**
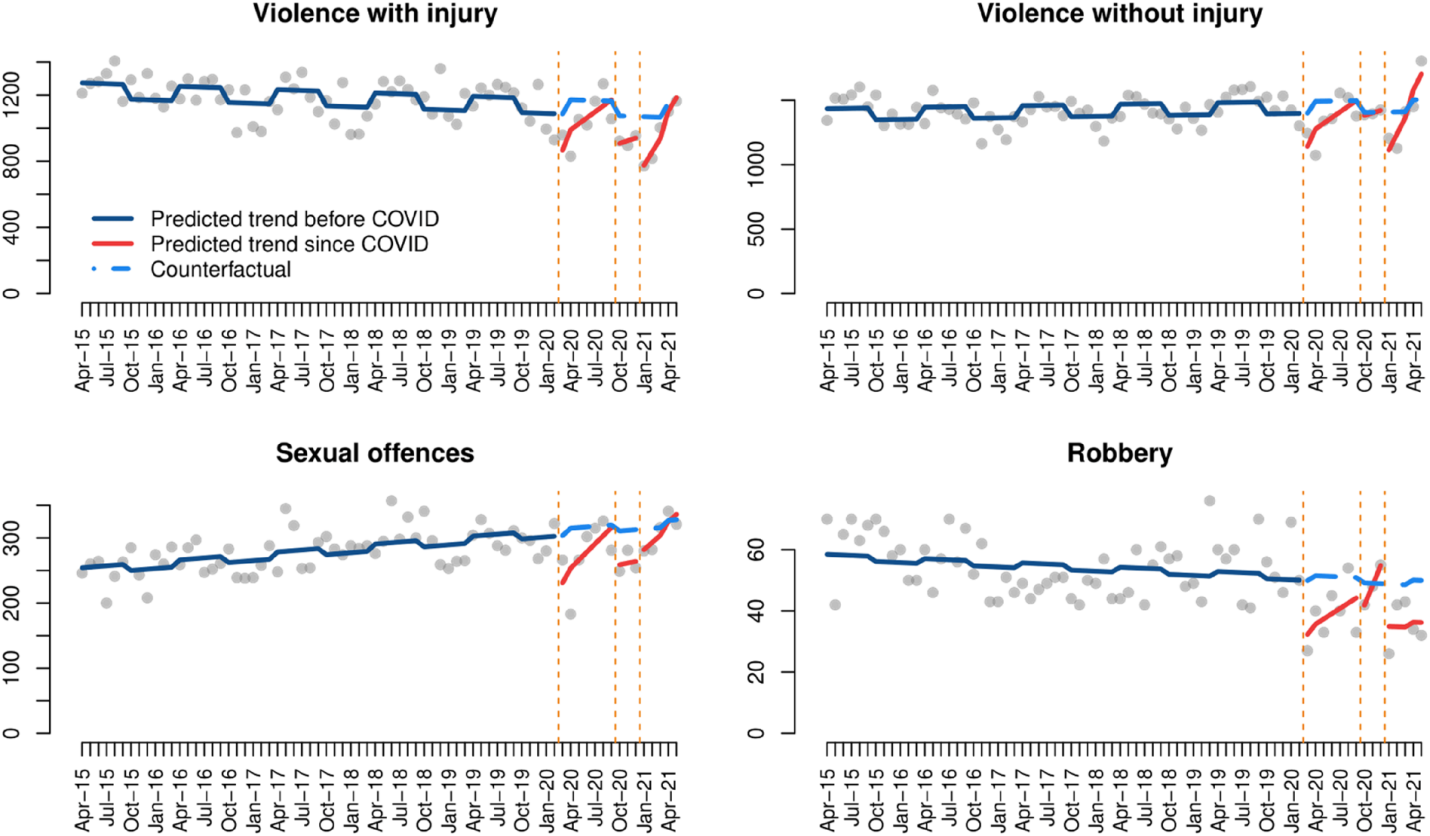
Interrupted time series analysis of violent and sexual crimes

The results of the ITS analysis of violence and sexual offences show that recorded crime levels decreased immediately after each COVID-19 lockdown, and then rapidly returned to pre-COVID levels (Fig. 3). The results of the ITS models, presented in Table 1, further reinforce this finding, showing that: (a) the immediate decrease in crime resulting from the first lockdown was statistically significant in all four cases; (b) the gradual increase in crime after the first lockdown is statistically significant in the case of violence with and without injury and sex crime, but not robbery; and (c) violent crime with and without injury significantly decreased immediately after the third lockdown, and returned to pre-COVID levels during the following months as lockdown restrictions were lifted.^4^

**Table 1:** Interrupted time series models of violent and sexual crimes

	Violence with injury	Violence without injury	Sexual offences	Robbery
(Intercept)	1186.9***	1342.5***	242.9***	56.9***
Time	− 1.7*	0.9	1.0***	− 0.1
First lockdown	− 257.7**	− 301.4***	− 84.2***	− 19.5*
Time since first lockdown	37.8*	43.2*	11.5*	1.8
Second lockdown	− 184.6	− 40.8	− 53.2	− 13.9
Time since second lockdown	18.2	18.1	1.5	6.6
Third lockdown	− 376.3***	− 419.4***	− 41.8	− 13.9
Time since third lockdown	82.3**	123.8***	10.0	0.0
Seasonality	88.0***	91.8***	10.3	1.7
Adjusted R^2^	0.51	0.35	0.30	0.31

***p-value < 0.001, **p-value < 0.01, *p-value < 0.05, ^+^p-value < 0.1

### Drug crimes, damage and public order

**Fig. 4:**
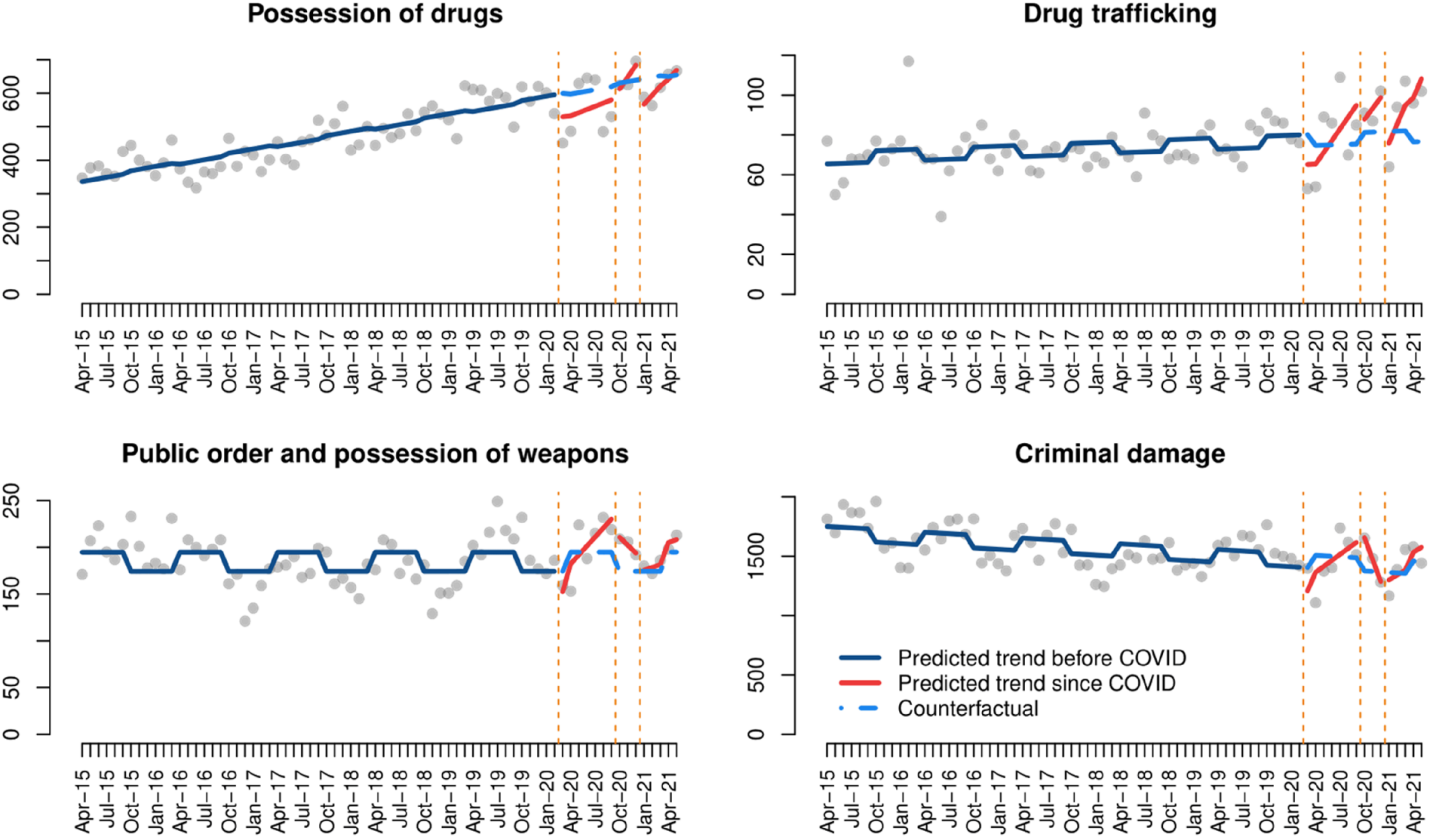
Interrupted time series analysis of drug crimes, damage and public order

Recorded drug-related crimes and public order/criminal damage offences show notably different trends. On the one hand, drug crime levels decreased immediately after each COVID-19 lockdown and progressively returned to pre-COVID levels during the following months. On the other hand, our analysis of criminal damage shows that crime decreased immediately after the first (and third) lockdowns, and then returned to the overall linear trendline, but the observed effect of the second lockdown was different to those seen above, showing a decrease in crime after October 2020. Changes in public order and possession of weapons offences were mainly driven by pre-COVID seasonal crime variation. This can be seen both in Fig. 4 and Table 2. The trend of criminal damage during the pandemic also follows remarkably similar patterns to pre-COVID trends, with seasonal increases during summer and lower levels in winter. Changes in criminal damage are related to both COVID-19 lockdowns and traditional crime seasonality. It should also be highlighted that drug trafficking offences are more frequent since COVID-19 than before.^5^

**Table 2:** Interrupted time series models of drug crimes, damage and public order

	Possession of drugs	Drug trafficking	Public order and possession of weapons	Criminal damage
(Intercept)	337.9***	71.0***	174.2***	1647.1***
Time	4.4***	0.2^+^	0.0	− 4.1***
First lockdown	− 75.2^+^	− 20.8*	− 31.5	− 250.3*
Time since first lockdown	5.1	5.7**	9.5*	54.1*
Second lockdown	− 48.7	1.2	45.1	464.0*
Time since second lockdown	31.1	5.3	− 8.5	− 181.9^+^
Third lockdown	− 99.1^+^	− 15.2	− 1.8	− 110.0
Time since third lockdown	22.7	9.3*	3.0	46.1
Seasonality	− 6.5	− 5.7^+^	20.6***	107.6**
Adjusted R^2^	0.76	0.29	0.19	0.36

***p-value < 0.001, **p-value < 0.01, *p-value < 0.05, ^+^p-value < 0.1

### Burglary

**Fig. 5:**
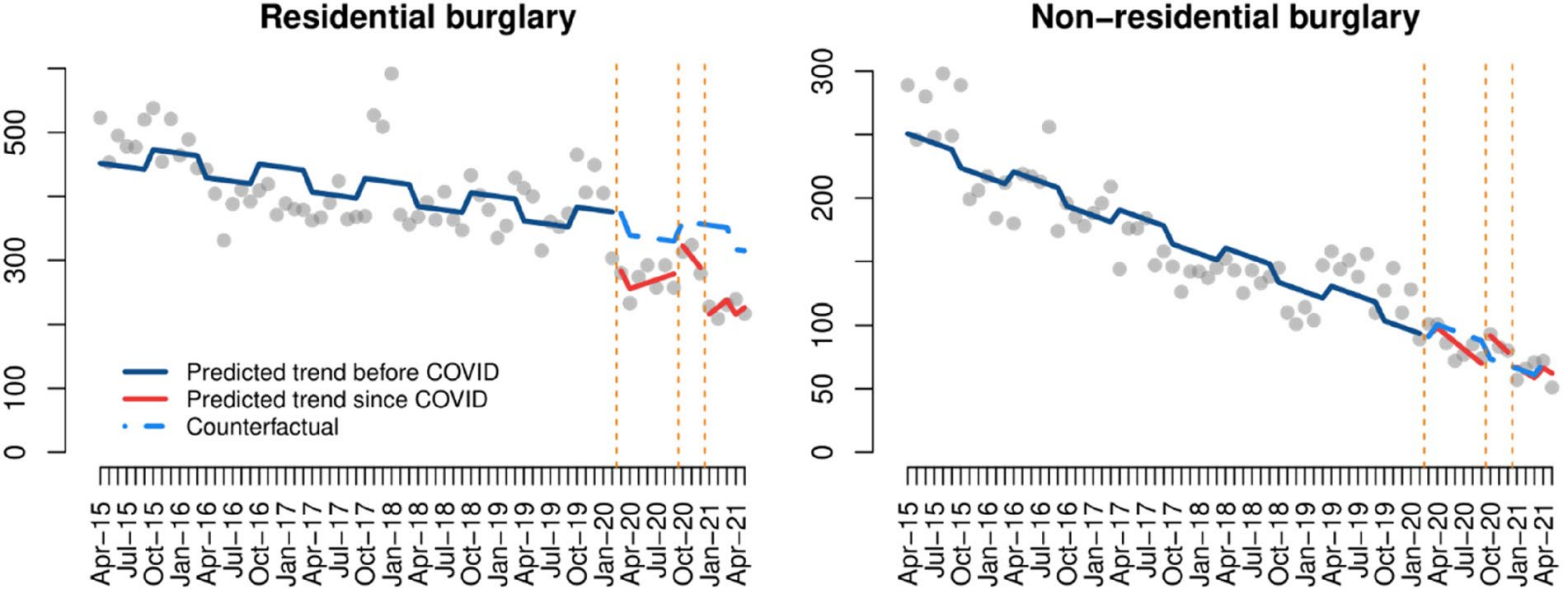
Interrupted time series analysis of burglary

There was a clear difference between the effect of COVID-19 on burglary trends when the crime occurred in residential dwellings in comparison to non-residential buildings. While residential burglary decreased after March 2020 and remained well below pre-COVID levels from then, non-residential burglary was not affected in any significant way by the COVID-19 lockdowns (Fig. 5). In the case of residential burglary, the segmented linear model results indicate that crime decreased immediately after the first and third lockdowns, and these changes were statistically significant (Table 3).^6^

**Table 3:** Interrupted time series models of burglary

	Residential burglary	Non-residential burglary
(Intercept)	486.1***	241.3***
Time	− 1.9***	− 2.5***
First lockdown	− 96.7*	3.9
Time since first lockdown	6.5	− 3.1
Second lockdown	− 23.0	22.3
Time since second lockdown	− 15.1	− 4.0
Third lockdown	− 151.4**	2.6
Time since third lockdown	12.7	− 1.7
Seasonality	− 32.6*	11.9^+^
Adjusted R^2^	0.66	0.84

***p-value < 0.001, **p-value < 0.01, *p-value < 0.05, ^+^p-value < 0.1

### Theft

**Fig. 6:**
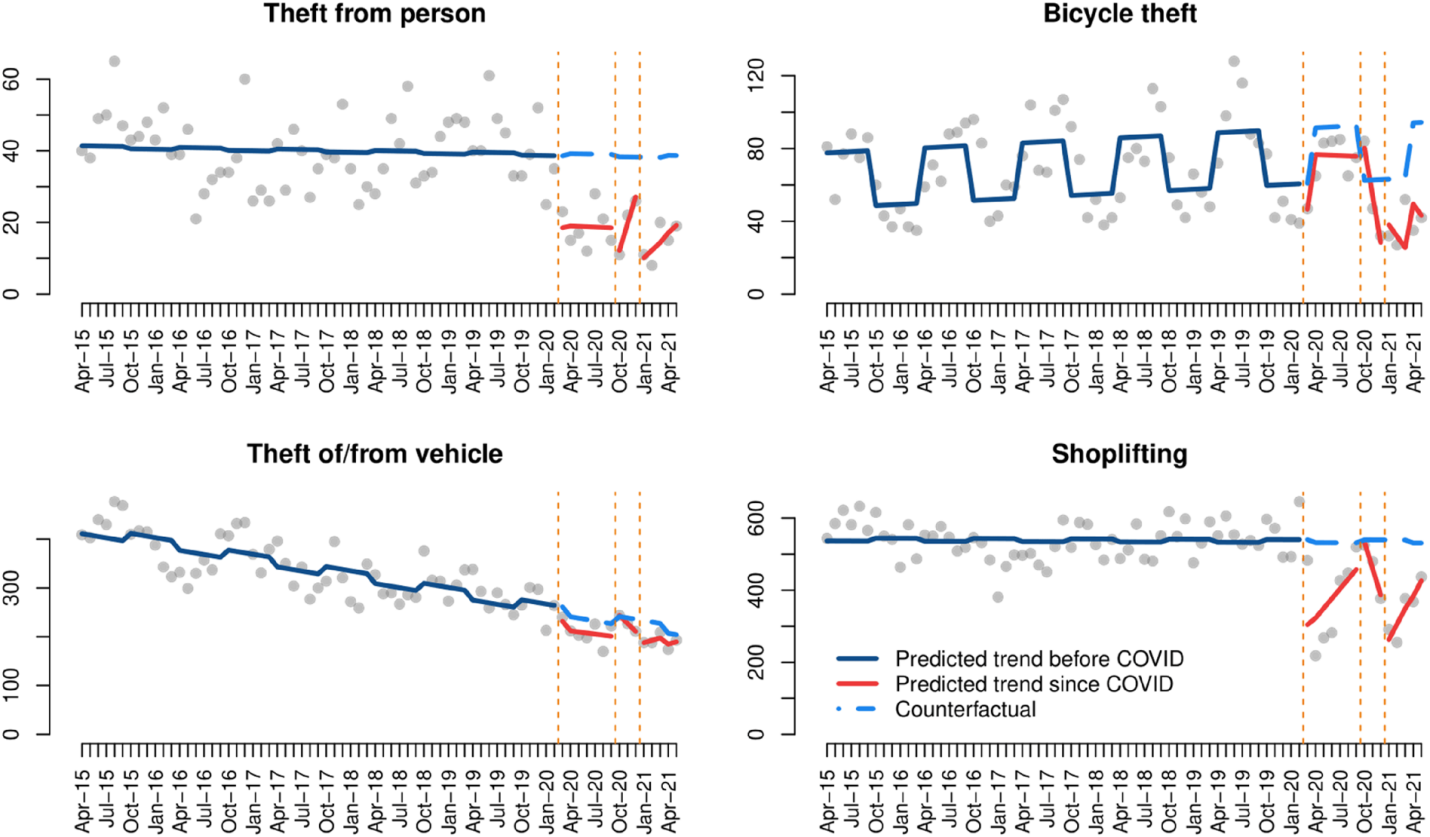
Interrupted time series analysis of theft

As can be seen in Fig. 6, there are important differences across the four types of theft analysed. First, reports of theft from persons decreased immediately after each COVID-19 lockdown, and then started to progressively return to pre-COVID levels after lockdown restrictions were lifted in each case. The ITS model in Table 4 indicates that the drops in theft from persons observed after each lockdown were statistically significant. Second, changes in bicycle theft during the pandemic appear to follow pre-COVID seasonal patterns, with large increases during summer and fewer crimes recorded in winter. However, the decrease in bicycle theft seen after the second lockdown provoked the lowest level registered since April 2015, and as such this decrease is statistically significant in the segmented linear regression model (Table 4). Third, the trend of theft of/from vehicles during the pandemic follows the steady decreasing trend seen before COVID-19, and none of the changes observed since March 2020 are statistically significant. And fourth, we observe that shoplifting experienced a substantial decrease after the first and third lockdowns, but subsequently started to bounce back to pre-COVID levels. Model results also show that, in this case, there was a decrease in crime records instead of an increase during the months following the second lockdown, and this does not appear to be attributed to pre-COVID seasonal trends. All these changes are statistically significant.^7^

**Table 4:** Interrupted time series models of theft

	Theft from person	Bicycle theft	Theft of/ from vehicle	Shoplifting
(Intercept)	40.8***	47.1***	431.6***	545.3***
Time	− 0.0	0.2^+^	− 2.8***	− 0.1
First lockdown	− 20.0*	− 13.9	− 30.1	− 263.2***
Time since first lockdown	− 0.1	− 0.4	0.6	27.0*
Second lockdown	− 33.7*	44.1	15.9	68.8
Time since second lockdown	7.5	− 26.2*	− 13.7	− 73.9^+^
Third lockdown	− 30.3**	− 18.6	− 53.1	− 319.6***
Time since third lockdown	2.2	− 6.5	7.7	43.1*
Seasonality	0.6	30.3***	− 17.6^+^	− 8.3
Adjusted R^2^	0.46	0.47	0.77	0.57

***p-value < 0.001, **p-value < 0.01, *p-value < 0.05, ^+^p-value < 0.1

### Fraud and cybercrime

**Fig. 7:**
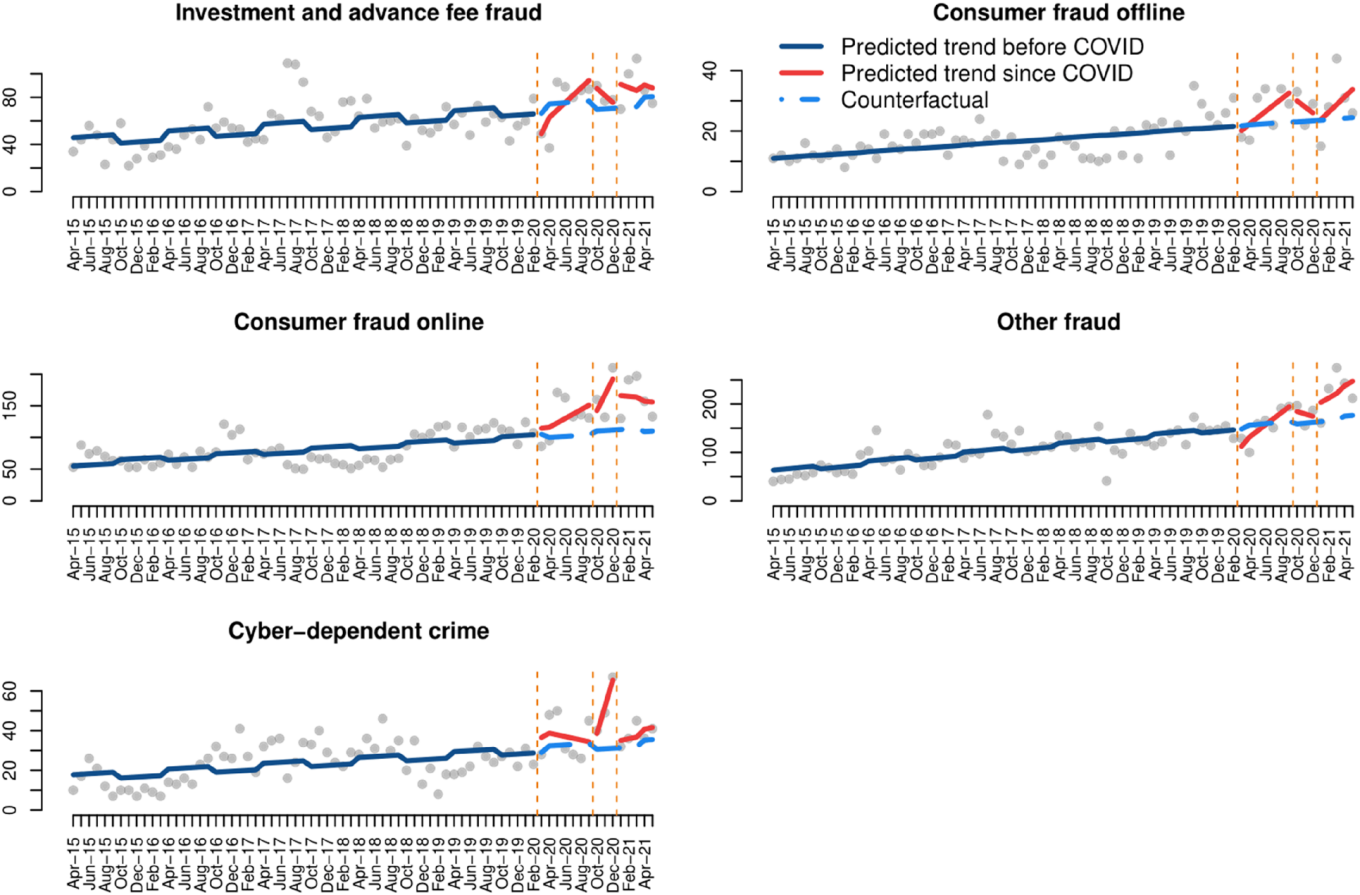
Interrupted time series analysis of fraud and cybercrime

We also analyse changes in fraud and cybercrime during COVID-19. At first glance, in Fig. 7, we observe a striking increase in recorded crime across all types of fraud, cyber-enabled or not, and cyber-dependent crime, since March 2020. In all cases there was also a steady increase since 2015, which is observed in the statistically significant effect of time on crime trends in the ITS model results (Table 5). There are, however, important differences across crime types.

**Table 5:** Interrupted time series models of fraud and cybercrime

	Investment and advance fee fraud	Consumer fraud offline	Consumer fraud online	Other fraud	Cyber-dependent crime
(Intercept)	37.7***	10.7***	59.7***	55.1***	14.4***
Time	0.5***	0.2***	0.8***	1.6***	0.2***
First lockdown	− 23.1	− 3.5	3.3	− 46.8*	8.7
Time since first lockdown	5.8^+^	1.9^+^	6.2	11.0*	− 1.1
Second lockdown	24.4	9.2	7.8	32.2	− 5.4
Time since second lockdown	− 6.5	− 2.2	24.2	-6.6	13.3*
Third lockdown	23.1	− 2.0	55.5*	32.0	3.0
Time since third lockdown	− 3.2	2.3	− 1.9	7.7	0.6
Seasonality	7.6^+^	0.1	− 5.5	6.8	3.1
Adjusted R^2^	0.38	0.48	0.67	0.74	0.46

***p-value < 0.001, **p-value < 0.01, *p-value < 0.05, ^+^p-value < 0.1

In the case of investment and advance fee fraud, which can be cyber-enabled in some cases but not others, recorded crime decreased immediately after the first lockdown, but there was an increase immediately after the second and third lockdown orders. None of these associations are statistically significant according to the results of the segmented linear model (Table 5). Regarding consumer fraud offline, the results of the ITS model show that the only temporal variable that may be statistically significant in explaining crime trends is the time since the first lockdown (Table 5), with a slight increase in crime during the months following March 2020. In the case of consumer fraud online, we see a steep increase in recorded crime during the COVID-19 pandemic, though the results of the ITS model (Table 5) show that the only statistically significant temporal variable was the immediate effect of the third lockdown. Regarding other frauds, which may be cyber-enabled or committed fully offline, we observe that the first lockdown may have provoked a decrease in crime which was then followed by an increase. Finally, with regards to cyber-dependent crime, we also see large peaks in crime levels during COVID-19, but the only variable with statistically significant effects is the time since the second lockdown, according to the ITS model (Table 5).^8^

## Discussion and conclusions

The COVID-19 pandemic and first lockdown orders led to rapid changes in everyday routine activities, which had direct effects on opportunities for crime (Nivette et al., 2021). Immediately after the first stay-at-home orders came into force in many countries in March 2020, crime researchers noted that various forms of property and violent crime had suffered a notable decrease due to the reduced opportunities for offenders to converge with targets in physical settings (Abrams, 2021; Ashby, 2020). Simultaneously, others highlighted that some forms of cyber-enabled and cyber-dependent crime had increased due to the growth in internet use for work and leisure (Buil-Gil & Zeng, 2021; Kemp et al., 2021; Lallie et al., 2021). After the first months of the pandemic, some of the social distancing restrictions were relaxed and rates of offline crime began to bounce back to pre-COVID trends (Balmori de la Miyar et al., 2021; Langton et al., 2021), but there is a gap in research about the mid-term effect of multiple lockdowns on online crime, and few previous studies have compared online and offline crime using the same dataset. To fill these gaps, the present paper analysed crime data recorded in Northern Ireland between April 2015 and May 2021 to analyse the short-, medium- and potential long-term effects of each lockdown on various forms of offline and online crime.

We identified that not all crime types were affected in the same way by the lockdown restrictions. Firstly, we observe that most forms of fraud and cybercrime rose rapidly during the early months of COVID-19 and continued growing up until May 2021. With the exception of drug trafficking, none of the traditional, offline crimes analysed above experienced clear increases during the pandemic, and thus fraud and cybercrime represent an exception to the overly simplistic view that crime decreased during COVID-19. The other crime type which also likely saw increases during the pandemic was domestic violence (Piquero et al., 2021), though some researchers note that it quickly returned to pre-COVID levels when lockdown restrictions were eased (Nix & Richards, 2021). Our data did not allow us to explore trends in domestic violence. In the case of fraud and cybercrime, there were notable differences across crime types. While recorded consumer fraud online, cyber-dependent crime and other fraud experienced notable growth from the first lockdown up until May 2021, a similar increase was not as clear in the case of consumer fraud offline, which decreased after some of the COVID-19 lockdowns. Investment and advance fee frauds, which can be cyber-enabled or not, appear to have decreased when the first lockdown came into place and possibly increased after that. It is possible, if not probable, that those forms of fraud that are enabled by digital technologies rose substantially during the pandemic (Buil-Gil et al., 2021), while non-cyber-enabled fraud suffered little variation or decreased (Kemp et al., 2021). The increase in everyday routine activities that people carried out online, often from under-protected home computers, including remote working, online shopping and online gaming, contributed to an almost-immediate increase in suitable targets on the internet during the months following March 2020. It is also probable that some ‘motivated offenders’ adapted their strategies to take advantage of new online opportunities, but many cyber-enabled and cyber-dependent offences require a set of technical skills which could not have been acquired in such a short time period. It is likely that the increase in cybercrime is due to the combined effect of a generalised increase in online targets of crime and a few offenders who successfully adapted to online opportunities during the pandemic, but further mixed-methods research should be conducted with offenders to analyse if there was a displacement of crime offending from offline to online environments. Moreover, we do not see any indication of cyber-enabled and cyber-dependent crime returning to pre-COVID trends. While our data only allow analysis of changes in crime up until May 2021, it will be important to study cybercrime trends during late 2021 and early 2022 to explore the possibility of a long-term increase in digital offences, which is of clear relevance for policy, practice and academic debate. As some have noted, the increase in online gaming, teleworking, meetings, online shopping and online dating may extend beyond COVID-19 (Nurse et al., 2021; Ofcom, 2021), thus creating new crime opportunities and accelerating the long-term upward trend in online crime.

Secondly, recorded violence, drug crimes and theft from persons experienced immediate drops when each of the three lockdowns came into force, and crime rates then returned to pre-COVID levels after lockdown orders were relaxed. These offences take place primarily in physical places that experienced decreases in mobility during each lockdown, and thus the opportunities for offenders to converge with suitable targets decreased with lockdown restrictions, and crime then returned to normal trends when social distancing measures were eased. Interestingly, while most forms of violent crime appeared to return to the same levels seen before the pandemic, drug trafficking not only bounced back to pre-COVID levels, but rates rose and remained well above those seen before the pandemic. Similar results were found in a study that analysed drug seizures over time in the United States (Palamar et al., 2021), and Langton et al. (2021) observed a peak in drug crime in England and Wales in May 2020, though a similar increase in drug crime was not observed in other countries (Balmori de la Miyar et al., 2021). It is still unclear whether recorded drug trafficking increased as a result of real growth in the supply of drugs or because law enforcement prioritised this type of crime during that period. Drug trafficking could have become more visible with less people walking the streets, or, alternatively, the rise in police recorded drug crime could be the result of greater police resources being dedicated to detecting these crimes. In contrast to violent crime and drug crime, recorded theft from persons decreased with each lockdown and then increased slightly, but rates in May 2021 were still below pre-COVID levels. Could this be because people who become involved in crime returned to the streets quickly after lockdown restrictions were lifted, but those not involved in crime did not leave the home as often as before COVID-19? That would explain why violent crime, in which two persons may mutually become involved in the incident, quickly returned to pre-COVID levels, while theft from persons, in which a crime target is needed, did not return to crime levels seen before the pandemic. That would also explain why residential burglary remained well below pre-COVID levels even in May 2021, due to the continued overall increase in the time spent by residents (capable guardians) at home. Further research is needed to identify changes in offender and victim activities.

And thirdly, some of the crime types with the most obvious seasonal patterns, including public order offences and possession of weapons, criminal damage, and bicycle theft, all of which tend to occur at much higher rates during summer, show a very similar seasonal variation during the pandemic. Crime records decreased with the first lockdown (March 2020) and increased during summer, after the second lockdown (October 2020) crime started to decrease during autumn and reached minimum levels with the stay-at-home order of the third lockdown in winter (January 2021), and after winter crime records began to increase again. Given the close correspondence between the traditional seasonal patterns in crime and the lockdown periods, it becomes difficult to fully comprehend the extent to which changes in crime are due to a continuation of pre-COVID crime seasonality or lockdown restrictions. In the case of criminal damage and bicycle theft, our model results—both the ITS and ARIMA coefficients—provide some support to the hypothesis that social distancing orders significantly affected crime trends, and thus we can expect that changes in crime are due to the combined effect of lockdown restrictions and seasonal variation. This is particularly evident in the case of bicycle theft, which displays a large, unusual decrease during the months following the second lockdown, when further restrictions related to the closure of cafes, hospitality and non-essential shops were introduced. The trend in shoplifting during the pandemic retains some similarities with that of bicycle theft, but both are markedly different from all other offline crimes. Shoplifting records suffered a very large drop immediately after both stay-at-home orders and progressively returned to pre-COVID levels during the following months, but with lower levels recorded by the end of the second lockdown (November/December) than when the second lockdown came into place in October. This is also likely to be the result of the stricter restrictions in place by the end of November, when cafes, hospitality and non-essential shops were closed. This is also shown in our ARIMA models, which indicate large decreases in both bicycle theft and shoplifting by the end of the second lockdown and with the third COVID-19 lockdown.

Our study also identified that not all COVID-19 lockdowns in Northern Ireland had the same effect on crime. The first lockdown, which was defined by a stay-at-home order and restrictions on all non-essential social and business activity, had an overall negative effect on most types of street crimes, due to a reduction in opportunities for the physical convergence between offenders and suitable targets. Similarly, the stay-at-home order of the third lockdown, in January 2021, had evident effects on mobility and crime opportunities. In contrast, the effect on crime trends of the second lockdown, which involved the closure of schools, universities and the hospitality sector but not a stay-at-home order, was less evident and non-significant in many cases. The trends in crime during the months following each lockdown also varied. In the case of the first and third lockdowns, most offline crime types suffered an immediate decrease and then progressively returned to the pre-COVID trend, while we observe the opposite trend during the months following the second lockdown. Records of some crimes, including bicycle theft and shoplifting, were lower by the end of the second lockdown, in November/December 2020, than in October. This is at least partly explained by the hardening of COVID-19 restrictions in late November, when the Northern Ireland Government imposed the closure of cafes, hospitality, non-essential shops and gyms. A similar pattern is observed in the case of fraud, where those fraud types that can take place offline suffered a decrease at the end of the second lockdown due to the additional social distancing measures, while online fraud experienced an increase in December 2020 due to the closure of shops and businesses and increased online shopping over the festive period.

While the findings presented in this article are first-of-its-kind and contribute to the criminological literature about the short-, mid- and long-term effects of rapid social changes on crime (offline and online), these are not free of limitations. The main threat to the validity of our findings is related to the use of police-recorded crime statistics as a primary source of data. Police-recorded crime data are known to be severely affected by measurement error arising from underreporting and underrecording, and it is yet unknown the extent to which the COVID-19 pandemic not only affected crime but also the measurement properties of crime statistics (Wallace et al., 2021). This may be particularly problematic in the case of cybercrime, given the low reporting rates that define these offences (van de Weijer et al., 2019). Future research is needed to explore if crime reporting and recording practices changed during COVID-19, thereby illuminating the extent to which research using police-recorded crime data to study changes in crime may be affected by measurement error. Moreover, due to data availability we analyse changes in crime across months, which may mask internal heterogeneity across days and weeks. Future research should analyse smaller temporal units of analysis where possible.

## Data Availability

All data and analytical codes are available from a Github repository (https://github.com/davidbuilgil/covid_crime_NI).

## Appendix

See Table

**Table 6:** Multivariate linear regressions with ARIMA errors (coefficients and 95% Confidence Intervals)

	Violence with injury	Violence without injury	Sexual offences	Robbery	Possession of drugs	Drug trafficking
First lockdown	− **113.8** [− 225.1, − 2.5]	− **157.2** [− 255.0, − 59.4]	− **77.0** [− 113.4, − 40.7]	− **28.2** [− 38.4, − 18.0]	− **53.3** [− 103.0, − 3.5]	− **30.2** [− 44.4, − 16.1]
Time since first lockdown	**41.9** [9.8, 73.9]	**24.9** [3.2, 46.6]	7.4 [-3.8, 18.6]	1.9 [− 1.0, 4.9]	9.1 [− 1.8, 20.1]	**5.2** [0.9, 9.5]
Second lockdown	− 134.5 [− 368.3, 99.2]	− 53.1 [− 183.7, 77.5]	− 68.8 [− 149.5, 12.0]	− **22.5** [− 44.2, − 0.8]	− 17.6 [− 105.5, 70.2]	8.6 [− 22.5, 39.7]
Time since second lockdown	40.4 [− 23.6, 104.5]	27.4 [-25.8, 80.6]	4.6 [− 14.6, 25.8]	**7.0** [1.1, 12.9]	**40.5** [5.4, 75.5]	4.1 [− 4.2, 12.4]
Third lockdown	− **321.2** [− 578.9, − 63.4]	− **453.0** [− 578.6, − 327.4]	− 54.4 [− 145.0, 36.3]	− **24.6** [− 48.6, − 0.6]	− 12.0 [− 86.7, 62.7]	− 14.2 [− 49.1, 20.6]
Time since third lockdown	**106.5** [65.8, 147.1]	**152.8** [117.2, 188.3]	13.3 [− 0.8, 27.3]	0.5 [− 3.3, 4.2]	**21.9** [4.9, 38.8]	**8.0** [2.6, 13.4]
Model components	(1, 1, 0)	(0, 0, 2)	(1, 1, 0)	(1, 1, 0)	(1, 1, 2)	(1, 1, 0)

	Public order and possession of weapons	Criminal damage	Residential burglary	Non-residential burglary	Theft from person	Bicycle theft
First lockdown	− **40.6** [− 67.4, − 13.9]	− **241.2** [− 397.3, − 85.1]	− **67.3** [− 123.6, − 11.0]	12.1 [− 11.0, 35.2]	− 14.5 [− 28.9, 0.0]	− 4.8 [− 24.0, 14.4]
Time since first lockdown	17.2 [− 4.2, 38.6]	**52.8** [16.2, 89.4]	0.2 [− 16.6, 17.0]	8.0 [− 10.7, 26.8]	0.4 [− 13.2, 14.1]	2.0 [− 2.5, 6.5]
Second lockdown	60.0 [− 83.9, 203.9]	**369.4** [93.2, 645.7]	2.4 [-119.4, 124.2]	84.1 [− 41.8, 210.1]	− 27.4 [− 117.7, 63.0]	**41.9** [11.1, 72.7]
Time since second lockdown	7.2 [− 23.4, 37.7]	− **186.0** [− 277.8, 94.3]	− 15.0 [− 47.1, 17.0]	2.6 [− 24.1, 29.3]	13.1 [− 8.0, 34.2]	− **27.5** [− 40.5, − 14.5]
Third lockdown	50.8 [− 163.7, 265.2]	− **295.4** [− 570.2, − 20.6]	− 109.4 [− 246.0, 27.1]	65.5 [− 122.4, 253.3]	− 7.7 [− 143.2, 127.9]	− **40.9** [− 68.6, − 13.3]
Time since third lockdown	17.0 [− 19.4, 53.4]	**73.5** [23.9, 123.2]	0.4 [− 20.7, 21.4]	14.2 [− 17.8, 46.1]	9.8 [− 21.7, 41.4]	3.3 [− 4.4, 11.0]
Model components	(2, 2, 0)	(0, 1, 1)	(1, 1, 0)	(2, 2, 0)	(4, 3, 0)	(1, 0, 1)

	Theft of/ from vehicle	Shoplifting	Investment and advance fee fraud	Consumer fraud offline	Consumer fraud online	Other fraud	Cyber-dependent crime
First lockdown	− 34.0 [− 74.8, 6.8]	**− 265.5** [− 308.7, − 222.3]	**− 22.5** [− 40.3, − 6.7]	− **24.2** [− 31.7, − 16.7]	**-127.2** [-151.8, − 102.6]	− 6.4 [− 39.5, 26.7]	**11.4** [3.6, 19.3]
Time since first lockdown	− 4.3 [− 13.8, 51.]	**12.6** [2.6, 22.7]	**7.8** [4.2, 11.4]	− 6.1 [− 14.6, 2.3]	**60.0** [30.5, 89.4]	21.6 [− 15.8, 58.9]	− 0.1 [− 1.9, 1.7]
Second lockdown	− 19.1 [87.9, 49.8]	90.6 [− 17.0, 198.2]	**33.5** [7.7, 59.5]	− 53.3 [− 108.4, 1.8]	**313.6** [120.7, 506.5]	85.9 [− 161.3, 333.2]	− 0.6 [− 14.4, 13.2]
Time since second lockdown	− 13.1 [− 37.5, 11.4]	− **103.6** [− 142.4, − 64.7]	− 4.9 [− 15.1, 5.3]	− **28.2** [− 45.2, -11.2]	− 3.8 [− 59.4, 51.8]	10.2 [− 41.4, 61.8]	**13.5** [8.5, 18.6]
Third lockdown	− **80.5** [− 145.2, -15.8]	− **407.3** [− 501.0, − 313.7]	19.8 [-2.8, 42.5]	− **158.4** [− 248.0, − 68.8]	204.7 [− 112.1, 521.5]	5.3 [− 338.9, 349.6]	6.5 [− 6.4, 19.5]
Time since third lockdown	0.7 [− 13.2, 14.6]	**36.2** [16.5, 55.9]	0.6 [− 5.0, 6.1	− 19.1 [− 49.5, 11.2]	7.3 [− 79.8, 94.4]	**100.5** [14.9, 189.0]	1.8 [− 0.7, 4.4]
Model components	(1, 1, 1)	(1, 2, 8)	(1, 1, 1)	(4, 5, 0)	(4, 5, 0)	(5, 4, 0)	(0, 1, 1)

6.

## Footnotes

1 Crime data in Northern Ireland can be accessed from the Police Service of Northern Ireland open data portal (https://www.psni.police.uk/inside-psni/Statistics/police-recorded-crime-statistics/) and the Open Data Northern Ireland portal (https://www.opendatani.gov.uk/dataset/police-recorded-crime-in-northern-ireland).

2 The first group includes violence with and without injury, sexual offences, and robbery. The second group includes possession of drugs, drug trafficking, public order and possession of weapons, and criminal damage (e.g., arson, forced entry into a property, graffiti). The third group includes residential and non-residential burglary. The fourth group includes theft from person, bicycle theft, theft of/from vehicle, and shoplifting. And the fifth group includes investment and advance fee fraud (when victims are asked to make upfront payments for goods, services, schemes or products that do not materialise; for example, fraud recovery scams, inheritance fraud, lender loan fraud, ‘419’ frauds, rental frauds, Pyramid schemes, pension scams or boiler room fraud), consumer fraud offline (for example, doorstep fraud and consumer non-investment fraud), consumer fraud online (for example, online shopping fraud, computer software service fraud and consumer phone fraud), other types of fraud (for example, fraud by abuse of trust, corporate employee fraud, credit card fraud, charity fraud, false accounting or business trading fraud), and cyber-enabled dependent crime (crimes that can only take place online, such as hacking, denial of service attacks and computer viruses).

3 We apply a variation of the Hyndman-Khandakar algorithm (Hyndman and Khandakar, 2008) to select the multivariate ARIMA error model with the best goodness-of-fit for each crime type. This algorithm seeks a data-driven selection of the components *p*  (order of the auto regressive model), *d* (order of differencing) and *q* (order of moving average), and *P*, *D* and *Q* (the seasonal components), of the model, thus finding the model that adjusts best to the data in each case. We evaluated the models selected using the Durbin-Watson and Ljung-Box tests to assess the autocorrelation of the residuals, and the KPSS test to assess the stationarity of fitted values. In some cases, the components of the model were adjusted manually to ensure that model assumptions were met.

4 The results of the multivariate models with ARIMA errors show similar results (see “Appendix”), but they also find that, in the case of robbery, the effects of the second lockdown (negative), time since second lockdown (positive), and third lockdown (negative) on crime are statistically significant.

5 The results of the segmented linear regression models (Table 2) are very similar to the multivariate ARIMA error regressions (“Appendix”), but there are some notable differences regarding the statistical significance of some temporal variables. For instance, the ARIMA error model shows that the negative effect of the first lockdown is also statistically significant in the case of public order and possession of weapon offences, the effect of time since the second and third lockdowns is also significant in the case of possession of drug crimes (while the negative effect of the third lockdown on possession of drugs may not be significant), and the negative effect of the third lockdown on criminal damage is statistically significant.

6 In the case of residential burglary, the ARIMA error model (“Appendix”) indicates that only the effect of the first lockdown was statistically significant.

7 The results of our ARIMA error models show similar results on the statistical significance of the changes in bicycle theft and shoplifting, whereas some differences are found in theft from person and theft of/from vehicle (see “Appendix”). First, the drops in theft from person after each lockdown are not statistically significant in the ARIMA error model. Second, the decrease in theft of/from vehicle after the third lockdown is shown to be statistically significant.

8 There are some differences between the results obtained from the ITS analysis and the ARIMA error models (see “Appendix”). In the case of investment and advance fee fraud, the results from the ARIMA error model show a statistically significant negative effect immediately after the first lockdown, and a positive effect in the period since the first lockdown and immediately after the second lockdown. The results of the ARIMA error model also show that the negative effect of the first lockdown, time since second lockdown and third lockdown on offline consumer fraud are statistically significant. In terms of consumer fraud online, the multivariate ARIMA error model shows that the time period after the first lockdown and the second lockdown were also likely to have statistically significant positive effects on online consumer fraud. The ARIMA error model results also show that the first lockdown had a negative effect on recorded online consumer fraud. With regards to other types of frauds, the ARIMA error model shows that only the increase from the third lockdown is statistically significant. Lastly, the results from the ARIMA error model further show statistically significant effects on the increase in cyber-dependent crime since the first and the second lockdown.
